# Metagenomic Insights into the Effects of Dietary Thymol on the Structure and Function of the Rumen Microbial Community in Beef Steers Consuming Forage

**DOI:** 10.3390/ani16060950

**Published:** 2026-03-18

**Authors:** Emma P. Fukuda, Yuan Lu, Emily Fowler, Russell W. Jessup, Merritt L. Drewery

**Affiliations:** 1 Department of Agricultural Sciences, Texas State University, 601 University Dr., San Marcos, TX 78666, USA; 2 Institute for Molecular Life Sciences, Texas State University, 601 University Dr., San Marcos, TX 78666, USA; y_l54@txstate.edu; 3 Translational Health Research Center, Texas State University, 601 University Dr., San Marcos, TX 78666, USA; prl55@txstate.edu; 4 Department of Crop and Soil Sciences, Texas A&M University, 400 Bizzell St., College Station, TX 77843, USA; rjessup@tamu.edu

**Keywords:** beef cattle, essential oils, plant extracts, thymol, shotgun sequencing, rumen microbiome

## Abstract

While essential oils are gaining momentum as a strategy to modulate rumen function and potentially reduce enteric methane in cattle, little is known about how their bioactive components, terpenes, affect rumen microbes. Our objective was to evaluate how in vivo doses of thymol affect the structure and function of the rumen microbial community via whole genome shotgun sequencing (WGS). Four beef steers were used in a 4 × 4 Latin square with four 28 d periods. Steers consumed ad libitum forage and received one of four thymol doses (0 [CON], 120 [120-T], 240 [240-T], and 480 [480-T] mg/kg forage intake). Rumen contents were separated into liquid and solid fractions, DNA was extracted, analyzed via WGS, and assessed with orthogonal contrasts. After FDR correction, no taxa were affected by thymol; however, raw *p*-values demonstrated responses to thymol supplementation for solid-associated uncultured *Lachnospiraceae* bacterium (*p* = 0.04), uncultured *Methanobrevibacter* (*p* = 0.05), and uncultured *Coriobacteriaceae* bacterium (*p* = 0.02). Liquid-associated uncultured *Prevotellaceae* bacterium (*p* = 0.03), *Prevotella* sp. (*p* = 0.04), and *Bacteroides* sp. (*p* = 0.02) also responded to thymol, with the highest abundances observed at various thymol doses. Genes involved in energy production and amino acid metabolism transport were observed at the highest abundances at 240-T, while genes associated with cell cycle control, cell division, and chromosome partitioning were present in the highest abundances at 120-T. The findings suggest that thymol exerts dose-dependent effects on rumen microbial abundances and functional pathways, with 240 mg/kg forage intake appearing to be the most effective dose to downregulate methanogenic enzymes while also enhancing the enzymes associated with metabolism without negatively impacting microbial diversity.

## Simple Summary

Essential oils (EOs) are fed to cattle to improve productivity and decrease methane production. Essential oils contain terpenes and terpenoids, both of which affect microbes in the rumen, the major digestive compartment of the stomach in cattle. It is important to identify how these terpenoids impact microbial populations to help producers maximize the impact of EOs on cattle performance. Some terpenoids, such as thymol, have strong antimicrobial effects, which could impact the animal’s ability to digest feed. To investigate microbial responses to EOs, we fed increasing amounts of thymol to steers and assessed changes in the rumen microbes. Feeding cattle thymol changed the rumen microbiome in dose-dependent ways. Most effects followed a curved (quadratic) pattern rather than a straight-line (linear) response. At moderate levels (240 mg/kg forage intake), thymol reduced genes linked to methane production and increased genes related to energy and protein metabolism, suggesting potential benefits for animal performance. Higher doses affected microbes differently, sometimes reducing beneficial groups. Overall, thymol appears to influence how rumen microbes function beyond just affecting which species are present, with a dose of 240 mg/kg forage intake striking the best balance between reducing methane potential and supporting microbial activity.

## 1. Introduction

Essential oils (EOs) have been researched as antimicrobial agents to modulate rumen microbial populations [1,2,3]. The antimicrobial effects of EOs are achieved via many structurally distinct terpenes that exert complex and unique actions that are not mechanistically well understood. Previous research investigating the efficacy of EOs as feed additives to improve ruminant animal performance is inconsistent [2,3,4,5] due to varying terpene concentrations within EOs that correspond with the environmental conditions in which a plant is cultivated [6,7]. Accordingly, the heterogenous nature of terpenes and their profiles within EOs has resulted in a conflicted body of literature on feeding EOs to ruminants [3].

Thymol is a cyclic monoterpene alcohol present in *Thymus vulgaris* (thyme) and oregano EOs. Thymol has a phenolic and low-molecular-weight structure that includes a hydroxyl group; these structural elements are likely responsible for the potent and non-specific antimicrobial activity of thymol [1,8,9]. Thymol, as with other terpenoids, primarily exerts antimicrobial effects by interfering with microbial cell membranes, which disrupts membrane conformation and stability and allows for the expansion and leaking of intracellular materials (i.e., ions, proteins; [10,11]). Although most bacteria use ionic pumps to prevent the loss of viability caused by a lowered trans-membrane ionic gradient, there is a significant energy requirement which negatively affects bacterial growth [8,12], thereby reducing population numbers and/or metabolites. In addition to interfering with cell membranes, thymol has also been observed to inhibit glucose utilization, transport, and growth in certain microbial species (i.e., *Streptococcus bovis*, *Selenomonas ruminantium* [13]). Further, dietary thymol alters certain rumen microbial species that are involved in the production of methane, ammonia, volatile fatty acids (VFAs), and lactate [12,13,14].

Much of the research that has investigated the impact of dietary EOs or terpenes on rumen microbial populations has been performed with amplicon sequencing (i.e., 16S rRNA and 18S). However, these sequencing strategies are limited by PCR bias and shallow sequencing depth, only providing reliable data at the genus level and omitting important species-level information [15]. Alternatively, whole-genome shotgun sequencing (WGS) facilitates analysis of complex changes in the microbiome composition at deeper taxonomic levels, which also allows for the characterization of functional aspects of microorganisms [16].

The objective of this study was to evaluate how graded doses of thymol affect rumen microbial community structure and function in vivo in beef steers consuming forage. Evaluating the ways in which known amounts of individual terpenes modulate rumen microbial populations and their function may elucidate the mechanisms that underlie the observed biological changes associated with feeding EO to ruminants.

## 2. Materials and Methods

Experimental procedures involving animals were approved by the Institutional Animal Care and Use Committee at Texas State University (#8693). Four ruminally cannulated Angus steers were used in a 4 × 4 Latin Square experiment where each steer was provided one of four treatments each period.

Steers (*n* = 4, 539 ± 54 kg BW) were housed in a partially enclosed barn in individual stalls and provided ad libitum water and a coastal blend of kleingrass and medio bluestem (3.4% crude protein [CP], 69.9% neutral detergent fiber [NDF]) at 130% of the previous 5 d average intake in addition to alfalfa cubes at 200 g/kg hay intake from the previous day. Treatments included a negative control with no feed additive (CON) and thymol at either 120 (120-T), 240 (240-T), or 480 (480-T) mg/kg forage (hay and alfalfa cube) intake from the previous day; this was dosed on an as-fed basis and was not adjusted for dietary dry matter (DM). These dose rates were comparable to levels of thymol supplemented in the form of thyme EOs in previous research [17].

Thymol was obtained in crystal form at 99% purity and stabilized using liquid nanocellulose to prevent volatilization. Each thymol dose was diluted with water to the target dose rate, soaked onto alfalfa cubes, and fed daily at 0730 h. Alfalfa cubes with thymol were completely consumed by steers; therefore, refusals were not collected.

Steers were adapted to stalls for 10 d before the experiment. Each period lasted 28 d with 8 d for treatment adaptation, 4 d for measurement of intake and digestion, 1 d for determination of ruminal fermentation and microbiome, and 1 d for sampling subcutaneous fat to determine accumulation of terpenes in adipose tissue. Finally, 14 d were allocated to account for carryover effect and allow for the microbial populations and terpenes in the adipose tissue to return to baseline; this was based on previous research that detected rumen microbiome shifts and stabilization after 14 days of dietary intervention [18]. During carryover, steers were fed ad libitum hay and 0.75 kg/d of soybean meal, but did not receive alfalfa cubes or thymol.

Ruminal contents were sampled (~1 kg) at 4 h after feeding on d 14 from four areas within the ventral and dorsal sac of the rumen for analysis of microbial community structure and function. Contents were mixed and squeezed through four layers of cheesecloth for liquid sample collection, and the remaining solid portion was mixed before sampling. Liquid and solid rumen environments were separated and analyzed separately, as it was established that these environments contain different bacterial communities [19,20], and liquid communities have a higher turnover rate in response to dietary changes over time [21]. Ruminal content samples were snap-frozen in liquid N and stored at −80 °C before thawing for subsequent DNA extraction and analysis.

### 2.1. DNA Isolation and Sequencing

Prior to sequencing, DNA was isolated from solid (*n* = 16) and liquid (*n* = 16) rumen samples using the QIAmp PowerFecal Pro DNA kit (Qiagen, Valencia, CA, USA) protocol and tested for quantity and quality using Qubit fluorometry. Purified DNA (*n* = 32) was used as a template for shotgun sequencing using the Illumina Novaseq platform (Illumina Inc., San Diego, CA, USA) with paired-end 150 bp reads. For data sharing, raw sequence data are available from the NCBI Sequence Read Archive under Bioproject PRJNA1417001.

### 2.2. Data Analyses

For analysis of microbial species, sequencing reads were mapped to two reference genomes: Hungate1000 Collection [21] and RUG2 ([22]; European Archive project ID PRJEB31266). The collections were downloaded from their respective data repositories, and contigs in each collection were concatenated in one file as a reference genome using custom perl scripts. For sequencing reads mapping, a genome index for each reference genome was made using software bowtie2 (V2.2.4) build function. Reads were aligned using Bowtie2 with default parameters, unless otherwise specified. No non-default alignment settings were applied.

Custom perl scripts were used to identify sequencing reads that uniquely map to one genetic loci without mismatch (alignment score = 0) or map to several loci, but one of the loci exhibits perfect sequence matching (alignment score = 0). To minimize cross-mapping among closely related rumen taxa and reduce inflation of relative abundance estimates, only reads exhibiting 100% sequence identity to a reference locus were retained for quantification [23]. Sequencing reads matching these conditions were quantified using custom perl scripts.

For microbial enzyme analysis, raw sequencing reads underwent quality control using fastp (v0.23.2) with adapter trimming and removal of low-quality bases (average Phred score < 30). Host DNA contamination was eliminated by mapping reads to the bovine genome using Bowtie 2 (v2.5.3). De novo contig assembly was performed with MEGAHIT (v1.2.9). Open reading frame (ORF) prediction on the assembled contigs was conducted using Prodigal (v2.6.3) with the -p meta option to account for metagenomic data. Predicted coding sequences < 99 bp were discarded. The remaining protein sequences were clustered at a 95% identity threshold using CD-HIT to generate a non-redundant protein dataset, retaining representative sequences. Functional annotation of the non-redundant protein dataset was achieved using eggnog-mapper (v2.1.12), aligning sequences against the Kyoto Encyclopedia of Genes and Genomes (KEGG) database (117.0), Clusters of Orthologous Groups (COG), and Carbohydrate-Active enZYmes (CAZyme). Custom Python (3.14.3) scripts were utilized to extract specific gene families, including methanogenesis-related genes, as well as proteins belonging to certain COG categories and CAZyme families from the functional annotation output. 

### 2.3. Calculations and Statistical Analysis

Statistical analyses were conducted in R statistical software (4.3.2). Read counts for each microbial strain were divided by total read counts and multiplied by 100% to generate relative abundance. Relative abundances of strains within a species were summed to determine species-level abundances. Read counts of COG categories and CAZyme families were also transformed into relative abundances.

Species-level relative abundances, COGs, and CAZymes were analyzed using the lmer model within the lme4 package, where fixed effects were treatment and period and the random effect was steer. All data underwent log_10_(x + 10^−6^) transformation followed by Shapiro–Wilk normality testing (retained if *p* ≥ 0.05). Linear and quadratic effects were determined using Type III Analysis of Variance (ANOVA), which is more robust for data without homogenous variances. Least squares means were calculated using the emmeans package (2.0.2) and separated using linear and quadratic polynomial orthogonal contrasts for graded doses of thymol. Benjamini–Hochberg false discovery rate (FDR) correction was applied across species and enzyme classes to control for Type I errors from the simultaneous testing of many taxa and enzyme-coding genes.

Heatmaps were generated using the heatmap3 package (1.1.9). Alpha diversity indices and strain richness were calculated using the VEGAN package (2.7-3). Quadratic and linear differences for alpha diversity were determined using the same model as described above.

Beta diversity was generated using a 3-dimensional non-metric multidimensional scaling (NMDS) technique in R with the Bray–Curtis method in the VEGAN package. The NMDS plot was built with the scatterplot3d package (0.3-45). The 3-dimensional version of NMDS was chosen to increase goodness of fit with a stress value below 0.1. Confidence ellipses were added using chi square analysis at a 95% confidence level with three degrees of freedom.

## 3. Results

Data related to the effect of thymol on diet utilization, fermentation parameters, and adipose tissue volatiles are published elsewhere [24]; data presented here were collected during the same project. Steers in the current study consumed a daily average of 5.66 kg total organic matter intake, which included the basal diet of hay (4.61 kg organic matter intake/d; 3.4% CP) and alfalfa cubes (1.05 kg organic matter/d; 17.9% CP; Table 1). Thymol was dosed based on mg/kg forage intake (hay and alfalfa cubes, not adjusted for dietary DM); steers receiving 120-T consumed an average of 854 mg thymol/d, 240-T consumed 1760 mg thymol/d, and 480-T consumed 3590 mg thymol/d. Across treatments, ruminal ammonia N averaged 2.17 mM, total VFA averaged 82.9 mM, and rumen pH averaged 6.6; there were not significant differences in these parameters across treatments [24]. Shotgun sequencing identified 4664 microbial strains that belonged to 221 species in either the liquid or solid rumen fraction (mean 90.3 ± 0.93 million paired-end reads/sample; range: 67.9–113 million).

### 3.1. Most Abundant Species

**Figure 1:**
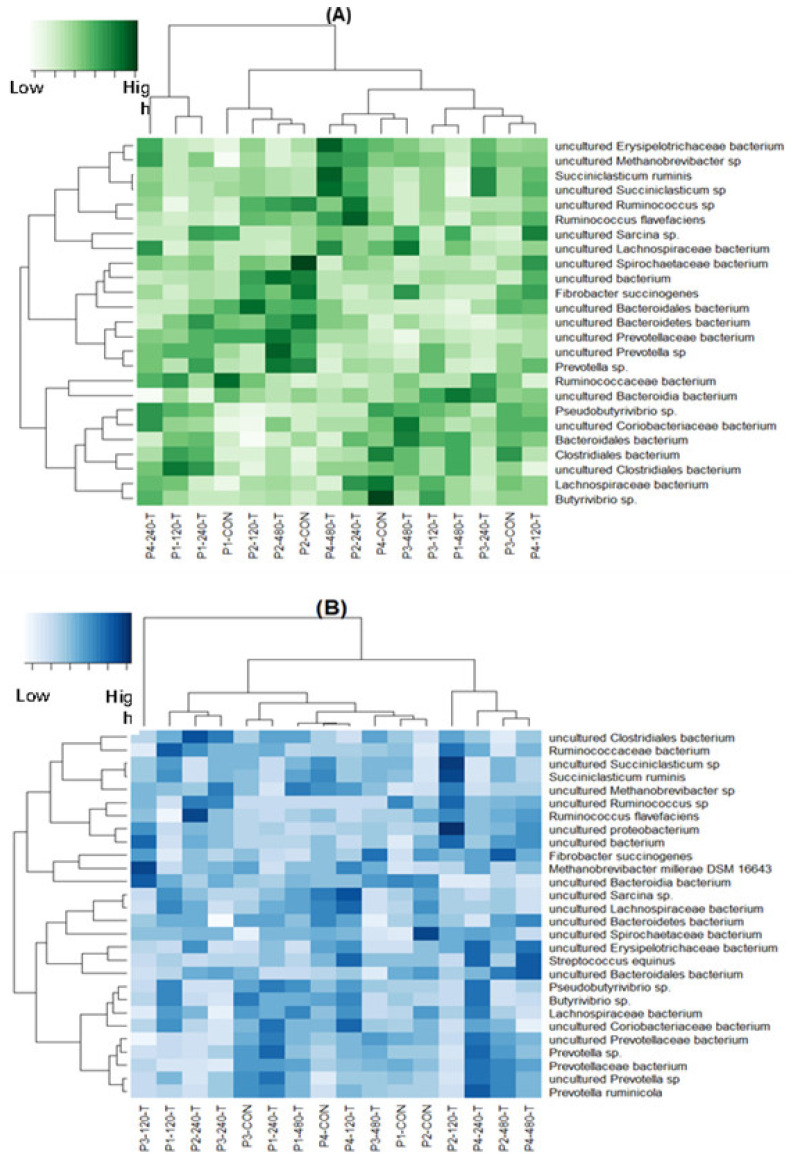
Heatmaps of the top 10% abundant species for liquid and solid rumen environments of steers consuming four doses of thymol. The *x*-axis indicates the treatment and period of the sample, where P1 = period 1 and treatments are indicated as CON (0 mg thymol); 120-T (120 mg thymol/kg forage intake); 240-T (240 mg thymol/kg forage intake); and 480-T (480 mg thymol/kg forage intake). The *y*-axis is the name of the microorganism species. (**A**) Solid rumen environment heatmap. (**B**) Liquid rumen environment heatmap.

The top 10% most abundant microbial species present in either the solid or liquid rumen fraction are shown in Figure 1. The solid rumen environment (Figure 1A) included, in order of abundance: uncultured *Bacteroidia bacterium* (19.13% ± 0.71), *Ruminococcaceae bacterium* (13.91% ± 0.43), uncultured *Clostridiales bacterium* (13.39% ± 0.36), uncultured *Prevotellaceae bacterium* (9.11% ± 0.44), uncultured *Lachnospiraceae* bacterium (8.33% ± 0.26), uncultured *Bacteroidales* bacterium (6.66% ± 0.30), uncultured *Methanobrevibacter* sp. (6.26% ± 0.30), *Butyrivibrio* sp. (2.62% ± 0.22), uncultured *Coriobacteriaceae* bacterium (2.09% ± 0.11), uncultured *Succiniclasticum* sp. (1.89% ± 0.09), uncultured *Bacteroidetes* bacterium (1.66% ± 0.11), uncultured *Sarcina* sp. (1.42% ± 0.08), *Fibrobacter succinogenes* (1.22% ± 0.15), *Prevotella* sp. (1.11% ± 0.06), *Ruminococcus flavefaciens* (1.01% ± 0.10), *Lachnospiraceae* bacterium (1.00% ± 0.03), uncultured *Ruminococcus* sp. (0.97% ± 0.03), uncultured bacterium (0.78% ± 0.09), uncultured *Prevotella* sp. (0.79% ± 0.06), uncultured *Erysipelotrichaceae* bacterium (0.55% ± 0.03), *Clostridiales* bacterium (0.53% ± 0.03), *Pseudobutyrivibrio* sp. (0.40% ± 0.03), *Bacteroidales* bacterium (0.31% ± 0.018), *Succiniclasticum ruminis* (0.23% ± 0.01), and uncultured *Spirochaetaceae* bacterium (0.17% ± 0.02). Therefore, 90% of the species present in the solid environment had an individual abundance of <0.1659%.

The liquid rumen environment (Figure 1B) included, in order of abundance: uncultured *Prevotellaceae* bacterium (18.00% ± 0.60), uncultured *Bacteroidia* bacterium (15.57% ± 1.03), uncultured *Bacteroidales* bacterium (13.18% ± 0.38), *Ruminococcaceae* bacterium (8.42% ± 0.45), uncultured bacterium (6.11% ± 0.40), uncultured *Clostridiales* bacterium (5.93% ± 0.24), uncultured *Methanobrevibacter* sp. (5.32% ± 0.40), uncultured *Bacteroidetes* bacterium (3.98% ± 0.16), *Prevotella* sp. (3.05% ± 0.12), uncultured *Prevotella* sp. (3.01% ± 0.17), uncultured *Succiniclasticum* sp. (2.18% ± 0.16), uncultured *Lachnospiraceae* bacterium (1.95% ± 0.12), *Fibrobacter succinogenes* (1.69% ± 0.19), uncultured *Ruminococcus* sp. (1.68% ± 0.20), *Butyrivibrio* sp. (1.33% ± 0.10), uncultured *Erysipelotrichaceae* bacterium (1.17% ± 0.10), uncultured *Coriobacteriaceae* bacterium (0.75% ± 0.06), *Lachnospiraceae* bacterium (0.45% ± 0.02), *Ruminococcus flavefaciens* (0.42% ± 0.03), uncultured *Spirochaetaceae* bacterium (0.37% ± 0.02), *Streptococcus equinus* (0.31% ± 0.04), *Prevotellaceae* bacterium (0.29% ± 0.01), *Prevotella ruminicola* (0.26% ± 0.01), uncultured *Sarcina* sp. (0.23% ± 0.03), *Succiniclasticum ruminis* (0.23% ± 0.02), *Pseudobutyrivibrio* sp. (0.21% ± 0.01), *Methanobrevibacter millerae* (0.17% ± 0.02), and uncultured *Proteobacterium* (0.16% ± 0.02). For the liquid environment, 90% of species present had an individual abundance of <0.1582%.

### 3.2. Alpha Diversity

There were no effects of thymol dose on strain richness or Shannon diversity for either liquid- or solid-associated rumen microbes (*p* ≥ 0.23; Table 2). For Simpson diversity, there was a linear increase for solid-associated microbial strains (*p* = 0.02), such that the lowest observed diversity was for CON (0.9983) and the highest for 120-T and 240-T (0.9988). For the total read count, liquid rumen microbial strains were quadratically affected by thymol dose (*p* = 0.02), where the lowest read count was observed for 120-T (*n* = 180,000,000) and the highest for 240-T (*n* = 236,000,000).

### 3.3. Beta Diversity

**Figure 2:**
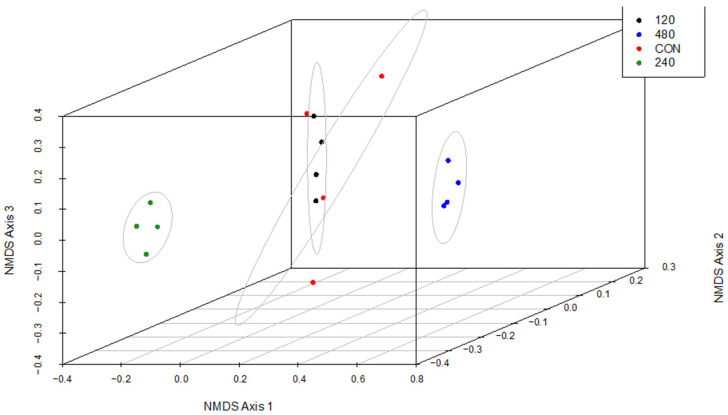
Three-dimensional non-metric multidimensional scaling (NMDS) plot comparing beta diversity of the solid-associated rumen microbiome of steers consuming doses of thymol. Steers consumed one of four treatments in addition to a basal forage diet: CON: control, 0 mg thymol/kg forage intake; 120: 120 mg thymol/kg forage intake; 240: 240 mg thymol/kg forage intake; 480: 480 mg thymol/kg forage intake. Confidence ellipses were drawn using chi square analysis with a confidence level of 95% and an alpha of 0.05.

For the beta diversity of solid-associated rumen microbes (Figure 2), there was no overlap between 240-T and 480-T (*p* ≤ 0.05), although 120-T and CON shared some overlap (*p* ≥ 0.05). Both 120-T and CON were different than 240-T and 480-T (*p* ≤ 0.05). The CON treatment had the widest spread, whereas points for the 240-T treatment were the most closely grouped.

**Figure 3:**
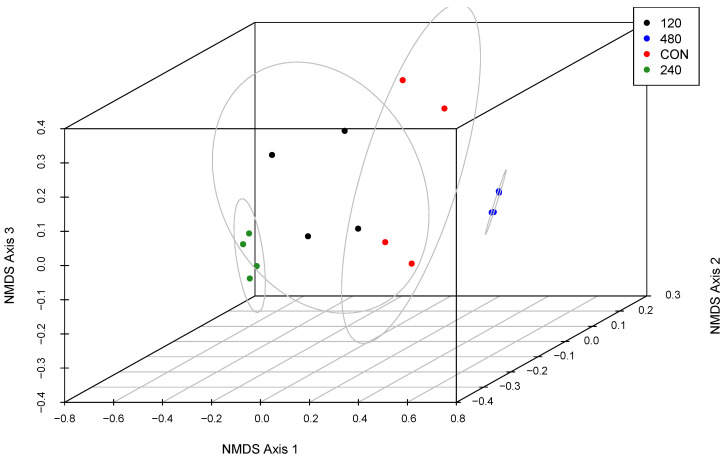
Three-dimensional non-metric multidimensional scaling (NMDS) plot comparing beta diversity of the liquid-associated rumen microbiome of steers consuming doses of thymol. Steers consumed one of four treatments in addition to a basal forage diet: CON: control, 0 mg thymol/kg forage intake; 120: 120 mg thymol/kg forage intake; 240: 240 mg thymol/kg forage intake; 480: 480 mg thymol/kg forage intake. Confidence ellipses were drawn using chi square analysis with a confidence level of 95% and an alpha of 0.05.

For the beta diversity of liquid-associated microbial strains (Figure 3), 480-T was different from other treatments (*p* ≤ 0.05) and was the most closely grouped. A beta diversity of 120-T was similar to 240-T and CON (*p* ≥ 0.05), and there were differences between 240-T and CON (*p* ≤ 0.05).

### 3.4. Species Abundance

Microbial species that were linearly or quadratically affected by increasing doses of thymol are outlined for solid-associated species in Table 3 and for liquid-associated species in Table 4, including the FDR adjusted *p*-values. After FDR correction, no taxa remained significant at q < 0.05. Although taxa did not meet significance thresholds when FDR was applied, several demonstrated nominal significance (*p* ≤ 0.05) for linear or quadratic contrasts when considering raw *p*-values. These data are presented to illustrate biologically relevant patterns that may warrant further investigation.

Uncultured *Lachnospiraceae* bacterium (*p* = 0.04; FDR corrected = 0.20) and uncultured *Coriobacteriaceae* bacterium (*p* = 0.02; FDR corrected = 0.19), high abundance (>1% relative abundance) solid-associated species, responded quadratically to thymol, with the highest abundances observed at 480-T. Uncultured *Methanobrevibacter* sp. also responded quadratically (*p* = 0.05; FDR corrected = 0.20), with the highest abundance observed at 240-T. For high-abundance liquid-associated species, uncultured *Prevotellaceae* bacterium (*p* = 0.03; FDR corrected = 0.42) and *Bacteroides* sp. (*p* = 0.02; FDR corrected = 0.42) responded linearly to thymol dose, with the lowest abundances observed at 120-T. Further, *Prevotella* sp. demonstrated a quadratic response, with the lowest abundance observed at 120-T (*p* = 0.04; FDR corrected = 0.66).

For solid-associated low abundance microbial species (<1% relative abundance), uncultured *Spirochaetaceae* bacterium (*p* = 0.02; FDR corrected = 0.20), uncultured *Pseudobutyrivibrio* sp. (*p* = 0.05; FDR corrected = 0.20), and *Treponema* sp. (*p* = 0.03; FDR corrected = 0.20) each responded quadratically to thymol, with the lowest abundances observed at 480-T. Quadratic responses were also observed for other solid-associated species, but with the greatest abundances observed at 480-T: *Peptostreptococcaceae* bacterium (*p* = 0.03; FDR corrected = 0.20), *Eubacterium pyruvativorans* (*p* = 0.05; FDR corrected = 0.20), *Lachnoclostridium citroniae* (*p* = 0.04; FDR corrected = 0.20), *Lachnoclostridium lavalense* (*p* = 0.03; FDR corrected = 0.20), *Sharpea azabuensis* (*p* = 0.01; FDR corrected = 0.15), *Blautia schinkii* (*p* = 0.04; FDR corrected = 0.20), *Megasphaera elsdenii* (*p* = 0.03; FDR corrected = 0.20), *Oscillibacter* sp. (*p* = 0.01; FDR corrected = 0.15), *Bifidobacterium boum* (*p* = 0.03; FDR corrected = 0.20), *Bifidobacterium merycicum* (*p* = 0.04; FDR corrected = 0.20), *Bifidobacterium pseudolongum globosum* (*p* = 0.05; FDR corrected = 0.20), *Denitrobacterium detoxificans* (*p* = 0.03; FDR corrected = 0.20), *Bifidobacterium thermophilum* (*p* = 0.04; FDR corrected = 0.20), *Selenomonas bovis* (*p* < 0.01; FDR corrected = 0.15), *Clostridium clostridioforme* (*p* = 0.04; FDR corrected = 0.20), *Ruminococcus bromii* (*p* = 0.05; FDR corrected = 0.20), *Enterococcus gallinarum* (*p* = 0.01; FDR corrected = 0.15), *Enterococcus faecalis* (*p* = 0.01; FDR corrected = 0.15), *Lactobacillus brevis* (*p* = 0.04; FDR corrected = 0.20), *Mitsuokella jalaludinii* (*p* < 0.01; FDR corrected = 0.07), uncultured *Methanobacteriaceae* archaeon (*p* < 0.01; FDR corrected = 0.15), *Enterococcus casseliflavus* (*p* = 0.01; FDR corrected = 0.15), uncultured *Ureaplasma* sp. (*p* = 0.05; FDR corrected = 0.20), *Clostridium innocuum* (*p* = 0.05; FDR corrected = 0.20), and *Enterococcus mundtii* (*p* = 0.01; FDR corrected = 0.15). There were also several solid-associated species that responded quadratically with the highest abundances at 240-T: uncultured *Chloroflexi* bacterium (*p* = 0.05; FDR corrected = 0.20), uncultured *Dialister* sp. (*p* = 0.03; FDR corrected = 0.20), uncultured *Denitrobacterium* sp. (*p* = 0.03; FDR corrected = 0.20), *Lactobacillus mucosae* (*p* = 0.01; FDR corrected = 0.15), *Pediococcus acidilactici* (*p* = 0.01; FDR corrected = 0.15), uncultured *Selenomonas* sp. (*p* < 0.01; FDR corrected = 0.07), *Proteiniclasticum ruminis* (*p* = 0.03; FDR corrected = 0.20), *Actinomyces ruminicola* (*p* = 0.05; FDR corrected = 0.20), and uncultured *Bifidobacteriaceae* bacterium (*p* = 0.02; FDR corrected = 0.19).

Low abundance liquid-associated microbial species that demonstrated either a linear or quadratic response to thymol were *Bacteroides* sp. (*p* = 0.02; FDR corrected = 0.42) and *Prevotella bryantii* (*p* = 0.01; FDR corrected = 0.66), both of which had the lowest abundances observed at 120-T. There were also linear responses for liquid-associated uncultured *Planctomycete* (*p* = 0.04; FDR corrected = 0.42), *Peptostreptococcaceae* bacterium (*p* = 0.05; FDR corrected = 0.46), *Sharpea azabuensis* (*p* = 0.04; FDR corrected = 0.42), *Bifidobacterium boum* (*p* = 0.04; FDR corrected = 0.42), *Denitrobacterium detoxificans* (*p* = 0.03; FDR corrected = 0.42), *Enterococcus gallinarum* (*p* = 0.01; FDR corrected = 0.42), and *Enterococcus faecalis* (*p* = 0.04; FDR corrected = 0.42), where the highest abundances were observed for 120-T with decreases as dose rate increased. Relative abundances of *Lactobacillus plantarum* (*p* = 0.04; FDR corrected = 0.42) and *Lactobacillus ruminis* (*p* = 0.03; FDR corrected = 0.42) also increased linearly in accordance with increasing thymol dose with the highest abundances observed at 240-T. Quadratic responses to thymol were observed for *Acetitomaculum ruminis* (*p* = 0.03; FDR corrected = 0.66) and *Lachnoclostridium clostridioforme* (*p* = 0.03; FDR corrected = 0.66), which were present in the lowest abundances at 480-T. *Blautia wexlerae* (*p* = 0.01; FDR corrected = 0.66) and uncultured *Denitrobacterium* sp. (*p* = 0.05; FDR corrected = 0.66) also quadratically responded to thymol, with the highest abundances observed at 120-T.

### 3.5. CAZyme, COG, and Methanogenic-Related Gene Abundances

**Figure 4:**
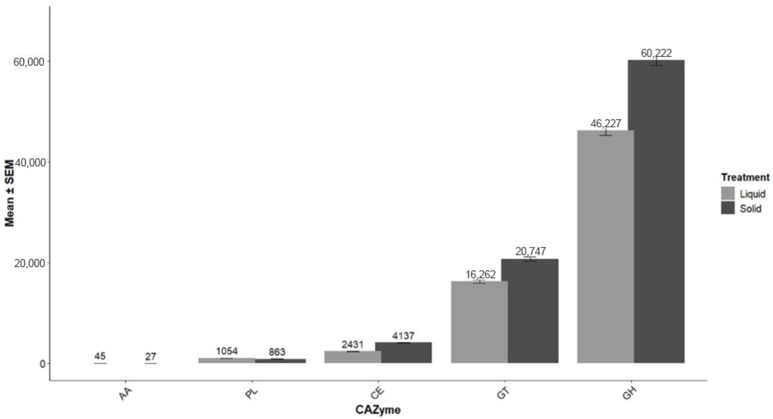
Carbohydrate-active enzyme (CAZyme) read counts for liquid (*n* = 16) and solid (*n* = 16) rumen environments averaged across treatments (*n* = 4) of beef steers consuming forage and supplemented various doses of thymol. Categories of CAZymes were AA = Auxiliary Activities, CE = Carbohydrate Esterases, GH = Glycoside Hydrolases, GT = Glycosyl Transferases, and PL = Polysaccharide Lyases.

**Figure 5:**
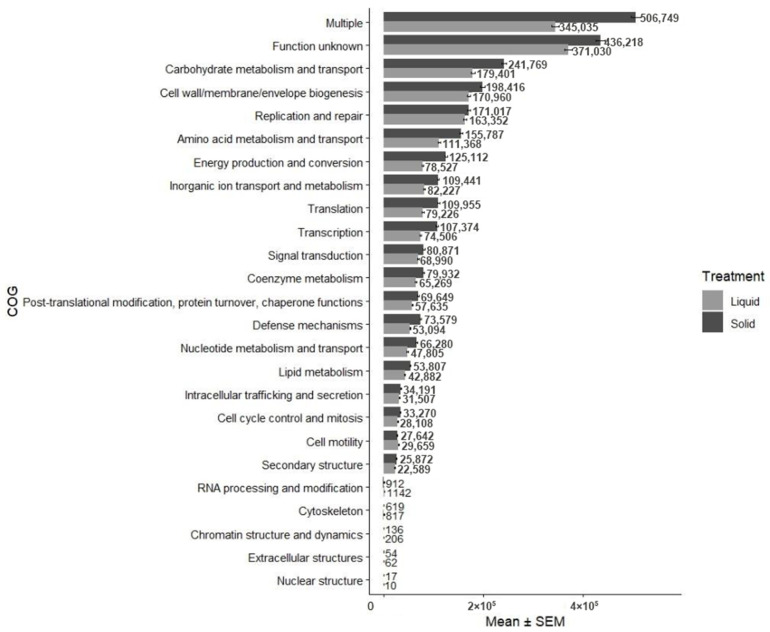
Read counts of clusters of orthologous genes (COG) in liquid (*n* = 16) and solid (*n* = 16) rumen environments of beef steers consuming forage and supplemented various doses of thymol.

Read counts of CAZyme and COG for categories not affected by treatment are presented in Figure 4 and Figure 5, respectively. Categories of CAZymes included auxiliary activities, carbohydrate esterases, glycoside hydrolases, glycosyl transferases, and polysaccharide lyases. Glycoside hydrolases had higher read counts than any other category (solid: 60,222; liquid: 46,227). Other than the categories “multiple” and “function unknown”, which had the greatest read counts, the COG category that had the highest read count was carbohydrate transport and metabolism, which was more represented in solid (241,769) than liquid (179,401) rumen environments.

**Figure 6:**
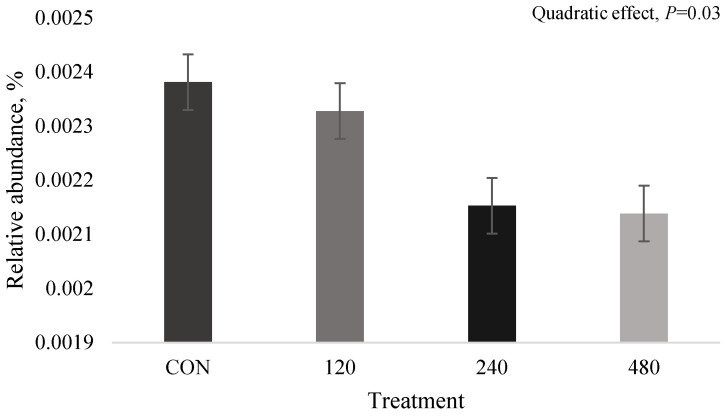
The effect of thymol on *mcrA* (EC 2.8.4.1; quadratic; *p* = 0.03) in beef steers consuming forage and supplemented various doses of thymol. Treatments are CON (0 mg thymol); 120 (120 mg thymol/kg forage intake); 240 (240 mg thymol/kg forage intake); 480 (480 mg thymol/kg forage intake). Data are displayed as mean ± SEM.

In the liquid rumen environment, *mcrA* (EC 2.8.4.1) quadratically decreased in accordance with thymol dose (Figure 6), with the lowest abundance observed for 480-T (0.0021%; *p* = 0.02; FDR corrected = 0.64), while other methanogenesis-related genes (i.e., mcrB, mcrC, mcrD, mcrG and pmoA) did not differ among treatments (*p* > 0.05).

**Figure 7:**
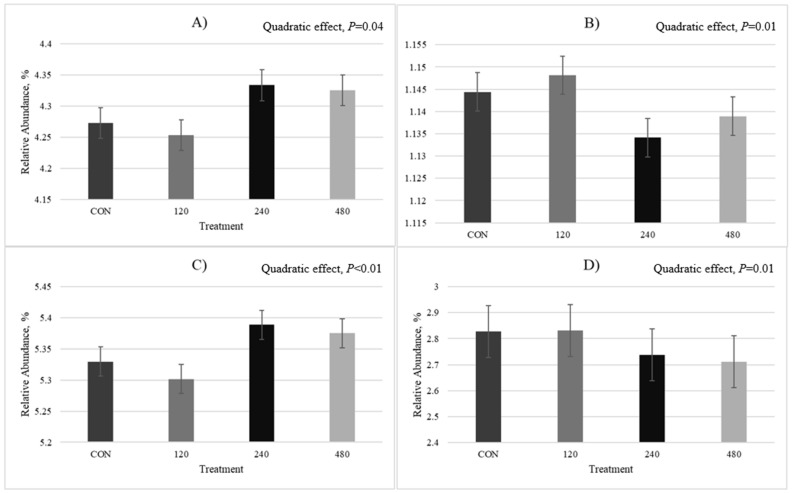
Clusters of orthologous genes (COG) enzymes significantly affected by thymol dose in solid rumen environment. Treatments are CON (0 mg thymol); 120 (120 mg thymol/kg forage intake); 240 (240 mg thymol/kg forage intake); 480 (480 mg thymol/kg forage intake). Data are displayed as mean ± SEM. (**A**) Quadratic effect of thymol on enzymes involved in energy production and conversion (*p* = 0.04). (**B**) Quadratic effect of thymol on enzymes involved in cell cycle control, cell division, and chromosome partitioning (*p* = 0.01). (**C**) Quadratic effect of thymol on enzymes involved in amino acid transport and metabolism (*p* < 0.01). (**D**) Quadratic effect of thymol on enzymes involved in signal transduction mechanisms (*p* = 0.01).

In the solid rumen environment, there were quadratic responses for numerous genes, including those involved in energy production and conversion (*p* = 0.04; FDR corrected = 0.28; Figure 7A), where the lowest abundances were observed for 120-T (4.25%) and the highest for 240-T (4.33%). Enzymes associated with cell cycle control, cell division, and chromosome partitioning quadratically decreased in accordance with thymol dose with the lowest abundance observed for 240-T (1.13%; *p* = 0.01; FDR corrected = 0.18; Figure 7B) and enzymes involved in amino acid transport quadratically increased (*p* < 0.01; FDR corrected = 0.05; Figure 7C), with the highest abundance observed for 240-T (5.39%). Thymol quadratically affected enzymes involved in signal transduction (*p* = 0.01), with a peak observed at 120-T (2.83%; FDR corrected = 0.18; Figure 7D).

## 4. Discussion

This was the first study to utilize WGS to assess the in vivo effects of graded doses of thymol on rumen microbial community structure and function in beef steers consuming forage. We employed a Latin Square design in combination with shotgun metagenomics, an experimental approach that is uncommon, likely due to the practical constraints associated with the resulting dataset. While Latin square designs are effective for controlling nuisance variation (e.g., steer and period effects), they do not increase sample size, which limits statistical power for high-dimensional shotgun metagenomic datasets that involve extensive multiple testing. Therefore, although no taxa or enzyme-encoding genes remained significant after FDR correction, we present and discuss raw *p*-values to highlight the biological patterns that may inform future hypothesis-driven research. Shotgun metagenomics provides richer functional information than amplicon sequencing approaches and, therefore, offers valuable insights to guide future research related to the interactions between thymol and the rumen microbiome.

Throughout the discussion, we reference data collected concurrently with those presented here, with a focus on the effect of thymol on diet utilization [24]. We have opted to publish those data separately from the current data due to the breadth and richness of our total dataset and because we did not attempt to explore the statistical relationships between diet utilization, including fermentation, and our metagenomics findings. However, as practical context is important and complements the current findings, we will reference that work in relevant portions of this discussion.

We observed that many fibrolytic, methanogenic, lactic-acid-producing, lactate-utilizing, and proteolytic microbial species responded to thymol supplementation, and diversity measures exhibited shifts at 240 and 480 mg thymol/kg forage intake. Further, several functional gene categories and enzyme groups—including those related to carbohydrate metabolism, methanogenesis, energy production, cell cycle regulation, amino acid transport, and signal transduction—exhibited quadratic responses to increasing thymol dose, with differences between the liquid and solid rumen environments.

The core rumen microbiome observed in this study aligns with previous research [25], which identified common species across ruminant animal breeds, species, geographical locations, and diets, and described the rumen microbiome as including genera *Prevotella*, *Butyrivibrio*, *Ruminococcus*, unclassified *Lachnospiraceae*, *Ruminococcaceae*, *Bacteroidales*, and *Clostridiales*. These genera were all represented in the top 10% of species in the current study in both the liquid and solid rumen environments, and each species within these genera had a relative abundance of >1%. The most abundant phyla in our study included Bacteroidetes, Firmicutes, and Euryarchaeota, similar to previous research in beef steers consuming forage [18]. This indicates that steers from our study had rumen microbial populations characteristic of forage-fed cattle, suggesting that the effects of thymol on the rumen microbiome are likely generalizable to other grazing cattle.

As in other research, the majority (90%) of rumen microbial species in our study were present at abundances <0.16% in both the liquid and solid environments; previous research demonstrates that these low-abundance (<1%) species have critical roles in microbial ecosystems [26,27,28]. Therefore, while several taxa identified in our study were present in low abundances, shifts in these microbial populations likely still have functional implications. Fermentation end-product profiles from the same experimental animals, reported previously [24], demonstrated treatment-associated changes in molar proportions of certain VFA and VFA ratios. These functional shifts are consistent with the microbial community restructuring observed here and provide complementary evidence that subtle taxonomic differences may translate into biologically meaningful metabolic outcomes. Further research could explore which low-abundance taxa drive the production of individual VFA, with a focus on taxa that affect the acetate-to-propionate ratio, given the functional relevance of these VFA.

Beta diversity analyses revealed shifts in diversity for animals receiving the highest dose of thymol for both solid and liquid rumen environments and for animals receiving 240 mg thymol/kg forage intake in the solid environment only. Therefore, the highest thymol dose provided in this study (480 mg thymol/kg forage intake) stimulated the greatest changes in rumen microbial composition.

Uncultured *Lachnospiraceae* bacterium, a carbohydrate-fermenting bacteria identified in the solid rumen environment, was present at higher relative abundances at increasing levels of thymol provision with a large effect size compared to control (Cohen’s *d* = 0.98). Many other cellulolytic, amylolytic, and hemicellulolytic species belonging to the phyla Bacillota, Spirochaetota, Actinobacteriota, and Bacteriodota also increased in relative abundance in accordance with the increasing rate of thymol supplementation, peaking at various doses. Few species associated with carbohydrate utilization were present in decreased abundances as thymol supplementation increased. Interestingly, enzymes involved in energy production and conversion were upregulated at our two higher doses (240 and 480 mg thymol/kg forage intake), with a large effect size observed for the 240 mg thymol/kg forage intake dose compared to control (Cohen’s *d* = 0.96), which may suggest the potential for increased energetic efficiency, although related research did not observe differences in diet utilization (i.e., intake and digestion) in the same experimental animals [24]. When taken together, these data suggest that thymol provided at 120–480 mg/kg forage intake likely do not inhibit abundances of carbohydrate-fermenting bacteria to an extent that would depress digestion and, thus, diminish the performance of beef steers consuming forage. The potential for thymol to enhance the energetic efficiency of ruminants should be further investigated.

We observed that the greatest abundances of proteolytic microbial species were generally observed at the highest dose of thymol (480 mg thymol/kg forage intake) with others at 240 mg thymol/kg forage intake dose. These observations align with a quadratic increase in the abundance of enzymes involved in amino acid transport and metabolism at 240 mg thymol/kg forage intake in the solid rumen environment exhibiting a large effect size compared to the control (Cohen’s *d* = 0.98). This contrasts findings of previous in vitro research, which documents that thymol decreased ruminal ammonia and accumulation of amino acids due to the inhibition of hyper-ammonia producing bacteria and, thus, deamination and proteolysis [1,29]. However, we did not observe treatment effects on ruminal ammonia–N concentrations in this same study [24]. Therefore, our data indicate that thymol does not inhibit proteolytic or hyper-ammonia-producing microbial species to an extent that interferes with ruminal N metabolism in beef steers consuming forage, in contrast with in vitro work [1,29].

As ionophores are banned for use as growth promoters and in other feed efficiency contexts in the European Union [30,31], there is growing interest in identifying alternative plant-derived compounds or other feed additives to modulate rumen fermentation without relying on antimicrobial compounds. Thyme EO and condensed tannins have been suggested as potential alternatives to ionophores [17]. However, condensed tannins may inhibit ruminal proteolysis and ammonia production; when sheep were fed forage containing 3% condensed tannins, relative abundances of certain proteolytic bacteria decreased [32]. As our findings suggest that thymol likely does not affect ruminal N dynamics, it may be a more attractive alternative for ionophores than condensed tannins. Previous research has reported that monensin specifically reduces Gram-positive bacteria [33], including those in the phylum Bacillota (previously known as Firmicutes); however, thymol in this study did not exert antimicrobial effects on a select group of microbes. The modes of action of monensin versus thymol deserve further investigation to assess the ability of thymol to favorably alter fermentation dynamics as ionophores.

Condensed tannins and ionophores often exert effects through decreasing the acetate-to-propionate ratio [33,34]. When supplementing monensin to beef steers, previous researchers observed shifts to microbial species that favor propionate production, translating to a decreased acetate to propionate ratio [33]. We observed that the relative abundances of certain propionate producing bacteria (i.e., *Prevotella* sp. and *Proteiniclasticum ruminis*) were highest when thymol was provided at 240 mg/kg forage intake, and *Prevotella bryantii* was highest at 480 mg thymol/kg forage intake. *Prevotella* sp. had a medium effect size at 240 mg thymol/kg forage intake compared to the control (Cohen’s *d* = 0.56). Further, lactate-producing bacteria, which convert lactate to propionate via the succinate pathway (i.e., *Selenomonas bovis*, uncultured *Selenomonas* sp., *Mitsuokella jalaludinii*, uncultured *Dialister* sp., and *Megasphaera elsdenii*), were generally present in the greatest abundances at 240 or 480 mg thymol/kg forage intake. We have previously reported that, in beef steers consuming forage and supplemented thymol at the same doses as utilized in this study, the acetate-to-propionate ratio quadratically increased in response to thymol supplementation and peaked at 240 mg/kg forage intake and then decreased at 480 mg/kg forage intake [24]. Based on these findings, we hypothesize that higher doses of thymol (i.e., >480 mg thymol/kg forage intake) may result in a decreased acetate-to-propionate ratio in cattle consuming forage, which would be undesirable from a performance standpoint.

Lactic acid bacteria (LAB) have many roles in the rumen, including producing bacteriocins, which are antimicrobial peptides that inhibit pathogenic rumen microorganisms [35]. Further, lactate production can serve as a precursor to propionate, which is a H sink, abating methanogenesis [36,37]. In our study, LAB belonging to the families *Enterococcaceae*, *Lactobacillaceae*, *Streptococcaceae*, and *Bifidobacteriaceae* increased in response to thymol, peaking at various doses. In the liquid rumen environment, many LAB were observed at their highest abundances at the lowest thymol dose (120 mg thymol/kg forage intake) and had decreased abundances at the two higher doses. Alternatively, in the solid rumen environment, the highest abundances of LAB were generally observed at the highest dose of thymol.

While LAB generally increased with thymol supplementation, which could contribute to excess lactate in the rumen [38], the abundances of many lactate utilizers also increased at 240 and 480 mg thymol/kg forage intake, which offers protection from acidosis when lactate production is high. Related research did not report acidic rumen conditions in beef steers supplemented thymol (120–480 mg/kg forage intake) to a forage diet [24]. However, we acknowledge that acidosis and, therefore, the potential for thymol to affect LAB, has more relevance in concentrate-fed versus forage-fed cattle (the focus of the current work). Accordingly, we recommend similar assessments to those described here in cattle consuming a starch-rich ration.

Relative abundances of the solid-associated methanogenic microbial species uncultured *Methanobrevibacter* sp. increased for steers supplemented thymol, peaking at 240 mg thymol/kg forage intake with a large effect size compared to control (Cohen’s *d* = 1.82), then decreasing at the highest dose of thymol (480 mg thymol/kg forage intake). While thyme EO has been documented to reduce methanogenic archaea and methane production [17], other studies assessing thymol in vitro have not observed changes in methanogenic archaea although methane production decreased with supplementation [1,39]. This indicates that thymol may inhibit methane production through means other than directly reducing methanogenic microorganisms. Our results support this as the *mcrA* gene, a well-established functional and phylogenic biomarker for methanogens [40,41] that is responsible for catalyzing the final step in methanogenesis, was decreased at the highest thymol dose in the liquid rumen environment, with a large effect size compared to the control (Cohen’s *d* = −1.19). However, as we did not directly assess methane production, we cannot confirm whether methanogenesis was reduced by thymol supplementation in our study.

It is worth stating that, although microbial groups categorized as “multiple” or “function unknown”, were not associated with changes in enzyme activities measured here, a lack of detectable effects does not imply a lack of functional relevance. Rather, the enzymatic assays conducted represent only a limited subset of ruminal metabolic processes, and these taxa may influence fermentation or other ruminal dynamics through alternative pathways not captured in our current analysis.

## 5. Conclusions

These data are limited by the combined experimental approach of a Latin Square design and a large metagenomic dataset, which reduced statistical power and prevented our results from meeting the FDR significance threshold. However, clear patterns were observed within this rich functional dataset, and we opted to discuss broad patterns based on raw *p*-values to highlight biologically meaningful responses that may guide future work. Our findings suggest that thymol exerts dose-dependent effects on rumen microbial abundances and functions, with 240 mg/kg forage intake appearing to be the most effective dose to downregulate methanogenic enzymes while also enhancing the enzymes associated with metabolism and without negatively impacting microbial community diversity.

This study was conducted in beef steers consuming forage, and further research is needed to determine the optimal thymol dose for cattle consuming concentrate-based rations. Other limitations of this study include a lack of direct measures of methane production measurements and, therefore, a reliance on the previous literature and the observed metagenomic functional patterns to assess the potential of thymol as an anti-methanogenic feed additive.

## Figures and Tables

**Table 1:** Chemical composition of basal diet ingredients ^1^.

Item	Hay	Alfalfa Cubes
**Chemical composition, % dry matter**		
Organic matter	89.59	86.73
Neutral detergent fiber	69.88	35.38
Acid detergent fiber	49.25	27.77
Crude protein	3.44	17.93
Calcium	0.35	1.71
Phosphorus	0.04	0.24
Potassium	1.27	2.87

^1.^ Basal diet consisted of ad libitum hay and alfalfa cube supplement supplied at 20% of the previous day’s total intake.

**Table 2:** Effect of graded levels of thymol on rumen microbiome strain richness and alpha diversity after rarefaction to 4000 sequences per sample in beef steers (*n* = 4) fed low-quality forage and alfalfa cubes.

	Treatment ^1^				SEM	Contrast *p*-Values	
	CON	120-T	240-T	480-T			
*n*	4	4	4	4		Linear	Quadratic
Richness							
Liquid	4664	4664	4665	4665	0.50	0.82	0.32
Solid	4663	4665	4662	4662	1.25	0.33	0.23
Shannon							
Liquid	7.34	7.31	7.32	7.29	0.03	0.68	0.42
Solid	7.29	7.29	7.30	7.29	0.03	0.76	1.00
Simpson							
Liquid	0.999	0.999	0.999	0.999	0.00	0.67	0.36
Solid	0.9983	0.9988	0.9988	0.9985	0.00	0.02	0.36
Total read count							
Liquid	220,000,000	180,000,000	236,000,000	224,000,000	9,460,118	0.12	0.02
Solid	216,000,000	217,000,000	216,000,000	226,000,000	18,432,174	0.84	0.68

^1^ CON: no supplement; 120-T: 120 mg thymol/kg forage intake; 240-T: 240 mg thymol/kg forage intake; 480-T: 480 mg thymol/kg forage intake.

**Table 3:** Linear and quadratic effects of thymol dose on the relative abundance of solid-associated microbial species in beef steers (*n* = 4) fed hay and supplemental alfalfa cubes.

	Treatment ^1^					Contrast *p*-Values		FDR ^2^	
	CON	120-T	240-T	480-T	SEM	Linear	Quadratic	Linear	Quadratic
*n*	4	4	4	4					
Microbial species ^3^	Relative abundance, %								
Uncultured *Lachnospiraceae* bacterium	8.0334	7.6286	8.4852	9.1717	0.385	0.28	0.04	0.97	0.20
Uncultured *Methanobrevibacter* sp.	5.4705	6.0890	7.3365	6.1438	0.411	0.09	0.05	0.97	0.20
Uncultured *Coriobacteriaceae* bacterium	1.9494	2.0318	2.1651	2.2230	0.088	0.48	0.02	0.97	0.19
Uncultured *Spirochaetaceae* bacterium	0.1969	0.1890	0.1586	0.1190	0.027	0.40	0.02	0.97	0.20
Uncultured *Chloroflexi* bacterium	0.0583	0.0665	0.0726	0.0719	0.004	0.20	0.05	0.97	0.20
*Peptostreptococcaceae* bacterium	0.0595	0.0599	0.0639	0.0641	0.002	0.82	0.03	0.97	0.20
*Eubacterium pyruvativorans*	0.0577	0.0576	0.0607	0.0616	0.002	1.00	0.05	1.00	0.20
*Lachnoclostridium citroniae*	0.0536	0.0541	0.0565	0.0576	0.001	0.83	0.04	0.97	0.20
*Lachnoclostridium lavalense*	0.0497	0.0502	0.0527	0.0535	0.001	0.76	0.03	0.97	0.20
*Sharpea azabuensis*	0.0488	0.0484	0.0508	0.0517	0.0008	0.60	0.01	0.97	0.15
*Blautia schinkii*	0.0472	0.0476	0.0498	0.0506	0.001	0.84	0.04	0.97	0.20
*Megasphaera elsdenii*	0.0460	0.0465	0.0489	0.0494	0.001	0.72	0.03	0.97	0.20
*Oscillibacter* sp.	0.0438	0.0449	0.0467	0.0482	0.0009	0.60	0.01	0.97	0.15
*Bifidobacterium boum*	0.0249	0.0248	0.0260	0.0265	0.0005	0.85	0.03	0.97	0.20
*Bifidobacterium merycicum*	0.0249	0.0247	0.0262	0.0265	0.0006	0.90	0.04	0.97	0.20
*Bifidobacterium pseudolongum globosum*	0.0246	0.0244	0.0257	0.0259	0.00004	0.82	0.05	0.97	0.20
*Denitrobacterium detoxificans*	0.0216	0.0217	0.0229	0.0231	0.0005	0.90	0.03	0.97	0.20
*Bifidobacterium thermophilum*	0.0202	0.0200	0.0210	0.0214	0.0005	0.83	0.04	0.97	0.20
*Selenomonas bovis*	0.0195	0.0193	0.0205	0.0209	0.0003	0.39	0.00	0.97	0.15
Uncultured *Dialister* sp.	0.0191	0.0192	0.0200	0.0198	0.0003	0.50	0.03	0.97	0.20
*Clostridium clostridioforme*	0.0133	0.0135	0.0140	0.0142	0.0003	0.73	0.04	0.97	0.20
*Ruminococcus bromii*	0.0124	0.0128	0.0130	0.0134	0.0003	0.52	0.05	0.97	0.20
Uncultured *Denitrobacterium* sp.	0.0111	0.0114	0.0123	0.0120	0.0003	0.57	0.03	0.97	0.20
Uncultured *Pseudobutyrivibrio* sp.	0.0123	0.0118	0.0113	0.0088	0.001	0.46	0.05	0.97	0.20
*Treponema* sp.	0.0121	0.0119	0.0104	0.0080	0.001	0.48	0.03	0.97	0.20
*Enterococcus gallinarum*	0.0100	0.0100	0.0105	0.0105	0.0001	0.88	0.01	0.97	0.15
*Lactobacillus mucosae*	0.0095	0.0095	0.0104	0.0103	0.0002	0.89	0.01	0.97	0.15
*Pediococcus acidilactici*	0.0091	0.0093	0.0098	0.0097	0.0001	0.31	0.01	0.97	0.15
*Enterococcus faecalis*	0.0086	0.0086	0.0091	0.0092	0.0002	0.91	0.01	0.97	0.15
*Lactobacillus brevis*	0.0076	0.0078	0.0082	0.0083	0.0002	0.58	0.04	0.97	0.20
*Mitsuokella jalaludinii*	0.0069	0.0068	0.0073	0.0075	0.0001	0.20	0.00	0.97	0.07
Uncultured *Selenomonas* sp.	0.0053	0.0053	0.0058	0.0057	0.00006	0.85	0.00	0.97	0.07
Uncultured *Methanobacteriaceae* archaeon	0.0038	0.0035	0.0049	0.0055	0.0004	0.25	0.00	0.97	0.15
*Proteiniclasticum ruminis*	0.0032	0.0031	0.0035	0.0034	0.0001	0.88	0.03	0.97	0.20
*Enterococcus casseliflavus*	0.0025	0.0025	0.0026	0.0027	0.00004	0.98	0.01	0.99	0.15
*Actinomyces ruminicola*	0.0025	0.0023	0.0028	0.0025	0.0001	0.43	0.05	0.97	0.20
Uncultured *Ureaplasma* sp.	0.0019	0.0021	0.0024	0.0035	0.0004	0.78	0.05	0.97	0.20
*Clostridium innocuum*	0.0022	0.0022	0.0024	0.0024	0.0001	0.81	0.05	0.97	0.20
*Enterococcus mundtii*	0.0014	0.0014	0.0015	0.0015	0.00002	0.55	0.01	0.97	0.15
Uncultured *Bifidobacteriaceae* bacterium	0.0013	0.0013	0.0015	0.0014	0.0005	0.63	0.02	0.97	0.19

^1^ CON: 0 mg thymol/kg forage intake; 120-T: 120 mg thymol/kg forage intake; 240-T: 240 mg thymol/kg forage intake; 480-T: 480 mg thymol/kg forage intake. ^2^ The Benjamini–Hochberg false detection rate (FDR) correction was applied across species to control for Type I errors. ^3^ Only microorganisms that were linearly or quadratically affected by treatment (*p* ≤ 0.05) are shown.

**Table 4:** Linear and quadratic effects of thymol dose on the relative abundance of liquid-associated microbial species in beef steers (*n* = 4) fed hay and supplemental alfalfa cubes.

	Treatment ^1^					Contrast *p*-Values		FDR ^2^	
	CON	120-T	240-T	480-T	SEM	Linear	Quadratic	Linear	Quadratic
*n*	4	4	4	4					
Microbial species ^3^	Relative abundance, %								
Uncultured *Prevotellaceae* bacterium	18.55	15.77	18.64	19.05	0.81	0.03	0.08	0.42	0.66
*Prevotella* sp.	3.00	2.67	3.31	3.22	0.167	0.18	0.04	0.60	0.66
*Bacteroides* sp.	0.14	0.11	0.15	0.15	0.008	0.02	0.04	0.42	0.66
Uncultured *Planctomycete*	0.08	0.11	0.09	0.08	0.007	0.04	0.13	0.42	0.66
*Prevotella bryantii*	0.05	0.05	0.06	0.06	0.001	0.03	0.01	0.42	0.66
*Peptostreptococcaceae* bacterium	0.029	0.031	0.031	0.029	0.0009	0.05	0.59	0.46	0.79
*Sharpea azabuensis*	0.027	0.029	0.028	0.026	0.0008	0.04	0.49	0.42	0.76
*Bifidobacterium boum*	0.014	0.015	0.015	0.014	0.0005	0.04	0.52	0.42	0.76
*Denitrobacterium detoxificans*	0.013	0.014	0.014	0.012	0.0004	0.03	0.09	0.42	0.66
*Acetitomaculum ruminis*	0.013	0.013	0.012	0.012	0.0003	0.44	0.03	0.80	0.66
*Lachnoclostridium clostridioforme*	0.010	0.010	0.0095	0.0094	0.0004	0.73	0.03	0.95	0.66
*Enterococcus gallinarum*	0.0065	0.0072	0.0072	0.0069	0.0002	0.01	0.30	0.42	0.70
*Enterococcus faecalis*	0.0063	0.0070	0.0070	0.0067	0.0002	0.04	0.41	0.42	0.74
*Lactobacillus plantarum*	0.0061	0.0069	0.0069	0.0064	0.0002	0.04	0.54	0.42	0.77
*Blautia wexlerae*	0.0061	0.0063	0.0058	0.0058	0.0001	0.45	0.01	0.79	0.66
Uncultured *Denitrobacterium* sp.	0.0064	0.007	0.0063	0.0054	0.0003	0.25	0.05	0.63	0.66
*Lactobacillus ruminis*	0.0025	0.0027	0.0027	0.0025	0.00007	0.03	0.81	0.42	0.91

^1^ CON: 0 mg thymol/kg forage intake; 120-T: 120 mg thymol/kg forage intake; 240-T: 240 mg thymol/kg forage intake; 480-T: 480 mg thymol/kg forage intake. ^2^ The Benjamini–Hochberg false detection rate (FDR) correction was applied across species to control for Type I errors. ^3^ Only microorganisms that were linearly or quadratically affected by treatment (*p* ≤ 0.05) are shown.

## Data Availability

The original data presented in the study are openly available in the NCBI Sequence Read Archive as Bioproject PRJNA1417001.
